# Post-pandemic occupational medicine and telemedicine in Portugal and France: what does the future look like?

**DOI:** 10.47626/1679-4435-2023-1194

**Published:** 2024-08-05

**Authors:** António Rui Marcelino Leal, Maria José Pinto da Costa, João Costa Amado

**Affiliations:** 1 Medicina Legal, Instituto de Ciências Biomédicas Abel Salazar, Porto, Portugal; 2 Centro de Investigação Interdisciplinar em Saúde, Universidade Católica Portuguesa, Porto, Portugal

**Keywords:** Portugal, France, occupational medicine, telemedicine, COVID-19, Portugal, França, medicina do trabalho, telemedicina, COVID-19

## Abstract

Based on personal experience over several years, we carried out a comparative analysis of two different European health systems, in Portugal and France, from a perspective comparing occupational medicine and use of telemedicine in a postpandemic context. This analysis addressed four aspects: Health System; Occupational Medicine; Telemedicine/Telework; and Future and Suggestions. The study employed searches and review of recent articles, guidelines, and recommendations from the authorities responsible for regulation (Medical Doctors Order, Labor Legislation, and Medical Collegiate Recommendations) and analysis of some statistical indicators from recent studies. Three tables on Occupational Health and Medicine present some relevant data and facilitate comparisons. Despite the difficulties of comparison, given the basic differences between these two systems (Beveridge *vs*. Bismark), it can be concluded that there is a greater acceptance of judicious use of teleconsultation in France (from 15 to 35%). This includes its use by occupational nurses, in the context of the “Visite de Information et Prevention”, with good acceptance among employers and employees. There are still some difficulties to be resolved concerning security, conducting biometrics, and objective examinations. We expect that these issues will be overcome with improved biosensing, adequate training, and proper regulation. Given the shortage of occupational physicians and the customary overrunning of legal deadlines, we believe that these possibilities and suggestions should be explored and adopted by the specialty’s Collegiates. Certain recommendations to this effect are made.

## Abstract (pt)

Com base na experiência pessoal de vários anos, foi realizada uma análise comparativa de dois sistemas de saúde europeus diferentes, de Portugal e da França, em uma perspectiva direcionada a uma comparação da medicina do trabalho e a utilização da telemedicina no contexto pós-pandêmico, em que ela se normalizou. Esta análise comparativa incide em quatro aspectos: sistema de saúde; medicina do trabalho; telemedicina/teletrabalho; e futuro e sugestões. Foram incluídas pesquisa e revisão documental de vários artigos recentes, normativas e recomendações das autoridades responsáveis pela tutela (Recomendações Colegiais, Ordem dos Médicos, Código do Trabalho), e foram feitas comparações de alguns indicadores estatísticos e estudos recentes. Três tabelas sobre a saúde, a medicina do trabalho e a telemedicina apresentam alguns dados relevantes e facilitam a comparação. Apesar da comparação difícil dadas as diferenças de base dos dois sistemas (modelo Beveridge *versus* Bismarck), parece ser possível concluir que existe uma maior abertura para a utilização criteriosa da teleconsulta na França (entre 15 e 35%), incluindo o seu uso por enfermeiras no contexto da *visite d’information et de prévention*, com uma boa aceitação pelos empregadores e trabalhadores. Ainda existem dificuldades por resolver, nomeadamente sobre a segurança e as dificuldades na sua utilização ou na realização da biometria ou exame objetivo. Por meio de melhorias no *biosensing* e de uma formação adequada, essas questões serão ultrapassadas. Dada a falta de médicos do trabalho e a habitual dilação dos prazos legais, essa possibilidade e as sugestões deveriam ser exploradas e enquadradas pelo Colégio da especialidade. Realizam-se algumas sugestões nesse sentido.

## INTRODUCTION

Stay-at-home policies adopted during the COVID-19 pandemic resulted in major increases in use of data transmission and improvement of the technologies involved,^1^ especially in richer countries, as echoed in scientific publications,^2^ and contributed to new business models in health care (B2B, B2C, B2B2C).^1^ The various different medical institutions adapted to the requirements imposed on them, giving birth to a new medical paradigm and, consequently, giving rise to an obligatory debate about the related legal, socioeconomic, and bioethical issues.^3^

In Portugal, Occupational Medicine (OM)still needs to address and overcome certain problems, such as a shortage of specialists, overrunning of legal deadlines, excessive reliance on supplementary tests and examinations to the detriment of analysis of real working conditions and their adaptation to the needs of workers (with disabilities, chronic illnesses, advanced age...) and, more recently, teleworking.

Notwithstanding, telemedicine has been legally regulated in Europe since 2011, by article 14 of eHealth Directive 2011/24/EU of the European Parliament and of the Council and by Commission Implementing Decision (EU) 2020/1023. France would like to promote a European health data space (within the TEHDAS [Towards the European Health Data Space] project), creating principles for data sharing between countries. This remains a growing tendency.

In this opinion article, based on our professional experience in France and Portugal, we present a comparative reflection on what is currently happening in these two European Union (EU) countries that have very different health care models.

## THE COMPARATIVE ANALYSIS

The differences between the two countries will be addressed with regard to four basic aspects and illustrated with three tables and indicators, followed by presentation of some proposals about the possible future of telemedicine in the specific legislative context of OM (TMLCOM).

### ON THE COUNTRIES AND THEIR HEALTH SYSTEMS (TABLE 1)

#### Portugal

According to the 2021 census, the population of Portugal was 10,344 million, of whom around 66% were of working age (15-64 years). Management of health service provision is attributed to three coexisting bodies: the National Health Service (NHS), financed by taxes, the Health Social Security System for certain professions (for example, the Institute for Disease Protection and Care), and private health care. The NHS offers universal coverage and tends to be free (a Beveridge type model), while around 25% of the population are covered by a voluntary insurance regime or subsystem.^4^ The system is currently underfunded and there is a shortage of general practitioners, with a consequent excessive demand for urgent and emergency services.

**Table 1:** General economic and health indicators for Portugal and France (summary of several indicators with quantitative comparison between the two countries)

	Portugal (Beveridge)	France (Bismarck)	Relative comparison (F/P)	References (origin and year)
Total population (in.)	10,297,081	67,488,449	6.5	Pordata, 2020
Per capita GDP	22,800 €	31,200 €	1.3	Pordata, 2020
Minimum wage	741 €	1,539 €	2.1	Pordata, 2020
Productivity/hour	65.8	126.5	1.9	Pordata, 2020
Life expectancy (years)	81.1	82.3	> 1.2	Pordata, 2020
Median age	45.5	41.9	> 4 years	Pordata, 2020
Workers per senior (> 65 years)	2.9	3.0	Similar	Pordata, 2020
Population with < 9 years’ education	44.6%	18.5%	0.4	Pordata, 2020
Physicians/100 thousand inhabitants	548.8	317.6	0.6	Pordata, 2020
Nurses/100 thousand inhabitants	756.1	1,167,3	1.5	Pordata, 2020
Employment rate	74.2%	72.1%	Similar	Pordata, 2020
Health care spending per inhabitant (2019)	2,314 euros	3,523 euros (EU)	1.5	OECD 2021^4^
Health care spending as percentage of GDP (2019)	9.5%	9.9% (EU)	0.4%	OECD 2021^4^
Ratio of physicians/nurses per 1,000 inhabitants	5.5/7	3.2/11 (F)	0.29 vs. 0.77	OECD 2021^4^

The basic reference was Pordata^©^ (year: 2020), which is based on a range of different sources, such as the European Association of Science Editors (EASE)/

European Central Bank (ECB)/European Commission/Eurostat/International Ergonomics Association (IEA)/International Labour Organization (ILO)/National Entities/

Organization for Economic Co-operation and Development (OECD)/United Nations (UN)/United Nations Economic Commission for Europe (UNEC)/United Nations

Educational, Scientific and Cultural Organization-Institute for Statistics (UNESCO-UIS)/World Health Organization (WHO). Some of these data were updated in July 2022. The last three indicators are from the European Health Observatory.^4^ F/P = France/Portugal; GDP = gross domestic product; EU = European Union.

#### France

France has a population and area that are seven times larger than those of Portugal, with a similar density of inhabitants per km^2^, and a higher mean life expectancy, despite a lower ratio of physicians to inhabitants, among other socioeconomic and health indicators^5^ (Table 1). The country’s health system is currently facing a crisis known as the “*déserts médicaux*”, which is a shortage of general practitioners in certain zones.^6^

France’s health care model is the Bismarck type (mutualized and interprofessional), and is free for people with chronic diseases (cancer, AIDS, and others), with disabilities, or who are considered “frail”. It is a complex system founded on multiple structures and many coparticipation systems (mutuals). Users are at liberty to choose their *médecin traitant* (a general practitioner who obligatorily refers), who enrolls them on their personal list, is responsible for follow-up, and is paid by the patients. The patient is later reimbursed by the Social System (caisses primaires d’assurance maladie [CPAM]), and also for supplementary tests and examinations, medications, and prostheses. Around 44% of physicians choose to be independent professionals, 32% work in hospitals, and 24% combine both.

Mutuals are obligatory for blue-collar workers and optional for others. Monthly payments are covered by employers and are part of the employment contract.

### ON OM (TABLE 2)

#### Portugal

Occupational medicine is a recent specialty, with a 4-year training period, including 256 hours of training, with obligatory and optional modules and internships at companies (directive No. 307/2012). In 2021, the Medical Doctors Order (MDO) had a total of 1,161 OM specialists registered, the majority of whom were male (58%) and approximately 45% of whom were over the age of 65.^7^

**Table 2:** Specific OM indicators in Portugal and France

	Portugal (Beveridge)	France (Bismarck)	Relative comparison (F/P)	References (origin and year)
No. of active workers (2021)	4,812,000	27,727,000	5.7	Pordata, 2020
Total number of firms (2019)	1,201,851	5,480,094	5.4	Pordata, 2020
Licensed OM firms	390	225	0.57	ACT tables (22) and www.presance.fr
Total no. of SOPs	1,161	4,812	4	MDO Table (2021)
Ratio of SOPs to 100 thousand inhabitants	11.6	7.2	0.62	Authors
Ratio of SOPs to total no. of firms in country	1,03	1,13	Similar	Authors
No. of workers followed up by SST	12,338	123,231	10	Authors
Price per year per worker (basic service)	20-45 euros	90-125 euros	4	Authors
No. of consultation hours	28 h (37 h × 75%)	21 h (35 h × 60%)	0.75	Authors
Mean consultations SOP/hour	4	2-3	Smaller	Authors

Where indicators specific to occupational medicine (OM) were unavailable, we compared certain indicators, extracted, or estimated, from prior databases or from personal professional experience.

WCA = Working Conditions Authority; SOP = specialist occupational physician; MDO = Medical Doctors Order; SST = *Santé et Sécurité au Travail*.

Specialists can be independent professionals (internal services), may work for public organizations or large companies, or may be attached to multiple or external services (business-to-business [B2B]). The last of these cases may be carried out in collective entities, generally small and medium for-profit enterprises that employ specialist occupational physicians (SOPs), health and safety technicians (HST), and occupational health nurses (OHN). These firms are licensed after a demanding candidacy process audited by the General Health Directorate/Working Conditions Authority(GHD/ WCA). They can offer services in clinics and/or mobile units and must have the equipment to perform tests and examinations such as electrocardiogram (ECG), respiratory function tests (RFT), hearing tests, and analyses.

SOPs are generally general practitioners and can be contracted as a freelance professional or via an employment contract and are habitually paid per hour and/or per consultation. Around 25% of their time should be dedicated to workplace interventions, management, analysis of workstations, etc. They should perform occupational fitness medical examinations every two years or annually (for those over the age of 50 or at high risk) and issue Medical Fitness Certificates (MFC), signed by the SOP, the employee, and the company’s Human Resources/HST. The ratios recommended by the GHD for OM are 1 hour/month per 10 high-risk employees or half that for low-risk workers (Law No. 102/2009).

In Portugal, patients may not work due to disease or accident after receiving a medical discharge. Once its term has expired, they return to work fulltime, and the SOP should adapt their tasks, their workstations, and their working hours to their pathology and/or their percentage degree of incapacity, which may be attributed by Medical Boards, according to the National Table of Disabilities (Law 352/2007).

The regulatory changes made by the College of OM during the health crisis did not change the total restriction on use of telemedicine, as happened in Brazil (article 2 of Federal Medical Council (Conselho Federal de Medicina [CFM]) resolution 2.183/2018, based on the same assumptions, but instituted use of personal protective equipment (PPI), barrier/isolation measures, and vaccination, among other measures.

#### France

Occupational medicine began with publication of the Health and Safety at Work Law (*santé et sécurité au travail* [SST]) (a foundational law from 1946) and was based on Forensic Medicine. It is based on articles R4121-1 to D4163-48 of the SST law.

Currently, SOP training in France also lasts 4 years, including 250 hours of theoretical teaching, eight semesters of training in hospital services, and several internships. As in Portugal, the mean age of SOPs is advanced (55 years), but there is a predominance of female SOPs, at 68%.

The labor legislation encompasses OM firms, which must obligatorily be social non-profit organizations, and with guaranteed equal management by employers and employees. After their candidacy has been validated (*agrément*) and a 5-year multiyear program has been approved (*contrats pluriannuels d’objectifs et de moyens* [CPOM]) by the *Directions régionales de l’économie, de l’emploi, du travail et des solidarités* (DRIEETS),^8^ these societies can conduct consultations, services, and tests and examinations, using multidisciplinary teams, including several technicians known as occupational risk prevention interventionists (*intervenants en prévention des risques professionnels* [IPRP]).

As in Portugal, there are autonomous and B2B services, based in clinics and/or mobile units. The standard supplementary tests and examinations in France are generally Visiotest, RFT, and hearing tests. They are the contractual responsibility of the SST firms themselves and are included in their standard annual fee structures. The French firms are 10 times the size of the Portuguese ones, in both dimension and response capacity (Table 2). According to the organization that represents them, there are 225 SST firms, with 17,600 workers and almost 15 thousand clinical centers and there is a trend towards mergers.

In France, according to the MDO, there is an obligation to exclusively practice OM (practicing curative medicine is forbidden), based on obligatory 35 h/week full-time (*contrat à durée indéterminée* [CDI]) or parttime (contrat à durée déterminée [CDD]) contracts, covered by a monthly remuneration table, and with career path possibilities.

Another difference between France and Portugal is founded on a Decree-Law from 27/12/2016 that changed the traditional system of SST consultations. The underlying principle is now an information consultation, renewable every 5 years and performed by a healthcare professional (SOP or OHN), known as an Information and Prevention Visit (*visite d’information et prévention* [VIP]).

If predefined risks are identified (asbestos, radiation, reproductivity-toxic substances …), the employee should be consulted by an SOP only, at intervals varying from 1 to 2 or 4 years, depending on the occupational risks and/ or individual characteristics (young people, people with disabilities, pregnant women…). The SOP should consult workers returning to work after absences exceeding 60 days because of disease or 30 days in the case of workplace accident (WA), within a maximum deadline of 8 days. The “pre-return” visit to prepare for return to work can be organized during the convalescence period.

The SOPs are responsible for coordinating the team of IPRPs, which includes the OHNs, the occupational health service assistants, the ergonomists, and the toxicologists,^8^ and also for carrying out several actions in the workplace (*actions sur le milieu de travail* [AMT]), occupying 33% of their working hours. These AMT are dedicated to workstation studies, company paperwork, and committee meetings, with a further 7% allocated to “*temps connexe*” (training, management, etc.). Therefore, 60% of their hours remain for consulting more complex cases, returnees, and people with disabilities to decide and guarantee unfitness for role status (common in France).

In 2009, Présanse decided to develop a system to standardize and define the different types of AMT in order to achieve uniformity of nomenclature and practices among the different SST services and facilitate statistical data collection. This process produced a standardized thesaurus (*thésaurus harmonisés*), that enabled systematic recording of AMT and other services, and which is revised annually and incorporated into OM software.

As in Portugal, SOPs do not have the power to prescribe the majority of supplementary diagnostic tests and examinations and/or treatments, but can recommend an internal consultation with a social worker (for prevention of occupational exclusion) and/or with a psychologist (for psychosocial risks), which are integral elements of the SST services.^8^ They can also make referrals for external hospital consultations.

Another important difference is the fact that the “Visio Consultation” (a TMLCOM consultation) is widely established and increasingly used, although it can be refused by the SOP/OHN. It was approved in Article L-3616-1 of the Public Health Law on July 27, 2019, and revisited in 2022 by the MDO.^9^

On 31 March, 2022, legislation was passed extending the responsibilities of SOPs to include counseling on exercise, nutrition, chronic pain, and addiction. The digital medical record (on-line) is now shared between the SOP and the general practitioner (*médecin traitant*). It also created mid-career and end-of-career consultations, which **Table 2.** Specific OM indicators in Portugal and France are yet to be regulated. The future trends envisioned for SOPs by the DRIEETS are reduced involvement in VIP consultations and increased involvement in return-towork consultations and sporadic examinations, with an increased use of VIP consultations, but conducted by the OHNs.^10^

The table used to calculate disabilities in cases of WA or occupational disease (OD) is called the “*concours médical*” (2001), which is used to determine a percentage of incapacity. If the level of incapacity is less than 50% and it is acceptable to the *médecin traitant* and Social Security, it may justify a situation that does not exist in Portugal, which is known as therapeutic part-time (*temps partial therapeutique*), for a maximum of 18 months. This status allows the worker to only work a percentage of their contracted working hours, with the remainder covered by the social security system. Definition of this percentage and adaptation of the workstation is the responsibility of the SOPs, who have the best knowledge of the true working conditions and have time allocated specifically for this task (AMT).

According to the Caisse Régionale d’Assurance Maladie d’Île-de-France, the main causes of OD in France are musculoskeletal injuries (91%), asbestosrelated diseases (6%), and conditions related to asthma/otorhinolaryngology (ORL) (3%), although undernotification is acknowledged.^10^

### ON TELEMEDICINE AND TELEWORKING (TABLE 3)

#### Portugal

Recognition and regulation of telemedicine in the NHS were defined on March 6, 2013, by Dispatch 3571/2013. In 2016, Ministry Council Resolution No. 67/2016 created the National TeleHealth Center.

**Table 3:** Specific OM and TMLCOM indicators in Portugal and Île-de-France (IF)

	Portugal (Beveridge)	France (IF) (Bismarck)	Relative comparison (F/P)	References/ year and notes
No. of workers	4,812 thousand	4,242 thousand (IF)	Similar	Pordata/DRIEETS^3^
Total number of SOPs	1,161	1,147 (IF)	Similar	MDO table (2021)/ DRIEETS (2021)
Ratio of workers per SOP or IPRP team	4,144	3,698	Similar	Estimate value
Use of TMLCOM	Not allowed	15 to 35%	Approximately 25%	DRIEETS^8^

To facilitate comprehension, we compared data for Portugal with data for Île-de-France, because of their similar population dimensions.

DRIEETS = *directions régionales de l’économie, de l’emploi, du travail et des solidarités*; IPRP = Intervenants sur la Prévention des Risques Professionnels; OM

= occupational medicine; MDO = Medical Doctors Order; SOP = specialist occupational physician; TMLCOM = telemedicine in the specific legislative context of occupational medicine.

The GHD regulations (guideline 010/2015) for teleconsultations define as obligatory informed consent, definition of the consultation as scheduled or urgent, clinical records, a final report (Conclusions and recommendations), and use of the International Classification of Diseases and Problems Related to Health (ICD-10).

The NHS already has a telemedicine system (“PDS Live”) in the implementation phase. The specialties that use it most are Radiology, Cardiology, Dermatology, Pathology, Psychiatry, and Rehabilitation.

The insurers took great advantage of this facility after the pandemic, coparticipating in teleconsultations, although the MDO set certain limitations as part of the prevailing regulations. The MDO Code of Ethics (D.R., Section 2, 139 and Reg. 707/2016) has included specific regulations for teleconsultations since 2016 (Chapter VII). It does not exclude any medical specialties, but places responsibility on the physician, who “is at liberty and entirely independent to decide whether or not to use telemedicine”, based on the assumption that it “must be conducted in conditions that are superposable to a consultation conducted in person and will only be employed when the physician has a clear and justifiable idea of the clinical situation”. However, how can conditions superposable with a consultation conducted in person be guaranteed without those involved being present?

Despite this, in Portugal, the data demonstrate elevated adhesion to teleconsultations during both waves of the pandemic, with a 44% utilization rate, reaching almost 2 million teleconsultations per month at the end of 2020.^4^

#### France

Considering its large dimensions, lower density of physicians, and higher number of nurses, in conjunction with better technological development, France adapted quickly to the pandemic and its issues.

The July 21, 2009, Law on hospital reform defined and regulated telemedicine for the first time in France. In 2018, this type of care was included in the general law regulating health insurance (*assurance maladie*), which started to reimburse for teleconsultations. The stay-athome orders provoked by COVID-19 caused an explosion in teleworking, driving the need for this modality,^2,3,11^ which began to be used in five different forms in France (Art. R.6316-1): teleconsultation, teleappraisal (teleinterconsultation), telesurveillance, telesurgery, and telescreening. Neither telediagnosis (a document-based medical opinion) or teleconsulting (between health professionals and administrators) are permitted, unlike in Brazil (CFM resolution of May 26, 2022).

The pandemic obliged the French to adopt these new modalities in regular check-ups by the *médecin traitant* (reimbursed at 25 €). A study initiated in 2020 investigating interest in telemedicine found that 68% of the French would be in favor of developing teleconsultation, with even higher rates among those under the age of 35 years (78%), from higher social classes (73%), and living in Paris (75%).^12^

On April 15, 2020, the Société Française de Santé au Travail (SFST) released a notification regulating OM activities during the pandemic, with a robust legal and contextual foundation.^13^ Several French firms producing software for OM began to incorporate systems for imaging/videoconsultation, while others began to offer external systems. Some firms invested in “biosensing”, creating telemedicine kits with equipment designed for specific contexts (for example, Navy, workplace, etc.). Still others invested in creation of dedicated cubicles or stations, with several biosensors, which were made available in pharmacies, supermarkets, or even in the workplace.

On the employers side, some companies (generally tech firms) included the SST’s capacity to conduct videoconsultation as an underlying condition of signing an OM contract, given the distance involved and the likelihood of teleworking (in many cases, fulltime). A study from 2021^9^ found that the reasons were reduction of travel costs, waiting, and administrative time, particularly when combined with sending MFCs SOPs/OHNs intended to continue using TMLCOM by e-mail with the SOP’s signature (the only signature regularly and 50% only occasionally, while 18% refused needed in France). This study also showed that 23% of to use it.

### ON THE FUTURE AND THE LIMITATIONS OF TMLCOM

Considering the current shortage of physician hours and the growing distances between patients and health professionals, telemedicine appears to be a consensual response in a post-pandemic era.^1,11,12,14^ Indeed, with respect to history-taking, there is no difference between digital communication and oral communication during a face-to-face consultation.

However, the same is not true of the objective examination, during which the physician must control vital parameters, auscultate, and “examine” the patient close up and using touch, which is not possible because of the current inability to mimic the sensitivity of the human hand, as has been recognized in Portugal for years.^15^ More specifically, if we consider that the great majority of OD are of a locomotor nature,^10^ this makes a functional musculoskeletal examination essential, to be able to determine the working tasks that the professional can execute.

Modern “biosensing” systems and wearable or implantable devices,^1^ whether by cable, contact, or wireless,^16^ enable easy execution of several types of biometry (ECG, RFT, hearing test…) under synchronous supervision by an SOP and/or OHN. This is possible as long as technical conditions are adequate (lighting, sound, definition...) and issues of data reliability and validity have been resolved.^11,16^ These supplementary tests and scheduled OM examinations can also be executed asynchronously by a trained professional on site (whether at work, home, or sports clubs), as is already done as part of home cardiovascular rehabilitation programs^17^ or in the Navy.^18^ This enables, for example, study of the worker’s physiological responses with cardiovascular conditions during a task involving intense effort or under adverse thermal conditions.

We therefore judge as indispensable development of new IT infrastructure, adequate training of those involved, and ethical and bioethical guidelines and standards for data recording and telemedicine care.^3,6,11^ Sharing patients’ digital medical records and accessing previous information/test results are other basic issues of TMLCOM that demand better interoperability between the different software packages, databases, and communication systems/protocols and secure data sharing between different entities (whether public or private) to avoid wasted time and loss of relevant information.

When firms (whether SST firms or the firms covered by them) permit teleworking, in accordance with collective agreements (a growing trend), they benefit from reductions in costs and in the need for space, in addition to enabling a better balance between family life and professional life, avoiding unnecessary absences in cases of mild diseases, outsourced care, or restrictions on movement (whether of the SOP/OHN of the worker).

It should also be noted that the progressive migration/internationalization of work in the EU and globally will force unification of medical care at a distance, as is already the case with foreign workers who have retired to Portugal, but maintain their physicians in France and/or vice-versa. In Brazil and China,^1^ because of their huge dimensions, this phenomenon already occurs between different states and regions, which is not the case in the EU, since an agreement on the European level is needed; one that encompasses jurisdictions. Will this be the probable advent of what we could call international telemedicine? What “national” laws and regulations and “territorial” limits should be imposed in these cases?

The need for good mastery of IT, compounded by problems related to bandwidth, internet outages, and access and interoperability difficulties are some of the obstacles to use of telemedicine.^1,3,9,11^ The advent of 5G internet and the introduction of Artificial Intelligence in medicine, which will be able to “anticipate” and/or substitute/supplement medical diagnosis, and the new definition of biometric/telemonitoring techniques and medical domotics (including home teleworking), will solve some issues and open new doors, while also raising complex bioethical questions.

Many issues still remain to be resolved, such as future legislation (which should be modified in response to rapid technological development), guaranteeing access security and interoperability between systems, changes to physical structures/hardware, coparticipation between subsystems, and new forms of payment.^3,11,16^ How should legislation be applied in the specific and legislative fields of OM? In this context, telemedicine will greatly facilitate compliance with legal deadlines for return to work (8 days) or amplify the importance of pre-return consultations, enabling telecommunication with the *médecin traitant* and/or the patient. These possibilities, with both advantages and limitations, should be reconsidered by the OM Collegiates.

It is also important to question the bioethics and logic of what can be called the “paradox of telemedicine”. In France, it is normally those with more qualifications, who are younger, more privileged, and more urban^12^ who most use it, when it should preferentially be serving more distant populations, with scant resources, displacement difficulties, and problems with access to health services, such as in rural environments^15^ or at long distances (Navy^18^), in contrast to what appears to be happening in China.^1^ There is, therefore, a need for a new mindset in Western medicine, which will undoubtedly be ensured by young physicians and by the gradual imposition of “digital and global health”.

## CONCLUSIONS AND FINAL SUGGESTIONS FOR TMLCOM

In synthesis and to underscore the prevailing practice of TMLCOM, we can state that, as a consequence of a health system with a different foundation, pandemic contingencies, the lower medical density, and the larger physical dimensions, France has adopted telemedicine in an optional manner, including for regular SST follow-up at utilization rates that vary from 15 to 35%^8^ (Table 3).

TMLCOM therefore appears to constitute a future opportunity for continued follow-up of workers, enabling improvements in telemonitoring of risk factors (occupational and/or personal) and in verification of true working conditions (in situ and on-line), and is a relevant factor for resource economy, facilitation of means, and worker satisfaction.

For this to occur, it must be accompanied by adequate regulation, limiting excesses in its use to the maximum possible. We offer here some operational suggestions to be pondered by the WHO/OM Specialty Collegiates: **•** It should be permissible to conduct up to one TMLCOM for each three or four in-person consultations, with all due records, documents, images and/or records attached (forensic medical proofs).

- TMLCOM should always be dependent on prior written consent digitally signed by the user and employer, explicitly setting out the ideal conditions, limitations/unforeseen circumstances, and respecting the General Data Protection Regulation, without which the physician would be exempt of responsibility in the event of any clinical error or technique failure.
- It should always be preceded or followed by an inperson consultation, at a time limit to be defined by the SOP, depending on the risks and specific clinical situation.
- Teleconsultations should always be validated by the SOP and conducted using certified equipment and by a duly qualified technician, under medical confidentiality, if there is a role for telebiometry (whether asynchronous or synchronous).
- A minimum level of training (6 hours?) should be guaranteed for any SOP and/or OHN who intend to employ telemedicine, which should be theoretical and clinical-practical (including practical cases).
- The videoconsultation should be aborted and an inperson consultation requested if the SOP realizes that the necessary conditions are not met.
- It should be the responsibility and even obligation of the *médecin traitant* to initiate arranging a teleconsultation, with or without the worker’s (virtual) presence, to define in conjunction with the SOP the best returnto-work (or pre-return) strategy (B2C model), which should be a future component of the OM *leges artis*.

Besides, if the disease is serious and requires hospital admission, the hospital physician should arrange this interconsultation between peers (hospital specialist, *médecin traitant*/*médecin généraliste en formation,* and SOP -B2B2C model), in order to coherently and promptly reduce the time taken to return to work, always ensuring medical confidentiality.

In the case of TMLCOM involving international teleworking and in cases such as Brazil and China,^1^ in which there is interaction between different states of the same country, the telemedicine firm should be licensed in the same country as the SOP/SST. Territorial limits and their legal implications (for example, AMT) should be covered in the workers’ contracts and explicitly included in the SST/SOP contracts.

This type of teleconsultation at a distance demands regular and random auditing by the MDO, in which case it becomes an effective and legalized method for improving worker integration and guaranteeing employee health and occupational quality of life, especially in the context of international teleworking.
